# Influence of Various Tea Utensils on Sensory and Chemical Quality of Different Teas

**DOI:** 10.3390/plants13050669

**Published:** 2024-02-28

**Authors:** Haowei Guo, Yani Pan, Chunlin Li, Yi Fu, Yanyan Cao, Qiang Chu, Ping Chen

**Affiliations:** 1 Tea Research Institute, College of Agriculture & Biotechnology, Zhejiang University, Hangzhou 310058, China; hwguo@zju.edu.cn (H.G.); yanipan@zju.edu.cn (Y.P.);; 2 Institute of Agro-Product Safety and Nutrition, Zhejiang Academy of Agricultural Sciences, Hangzhou 310021, China; chunlinli0304@163.com; 3 Department of Cultural Heritage and Museology, Zhejiang University, Hangzhou 310028, China

**Keywords:** tea utensils, material, different tea, flavor characteristics

## Abstract

The choice of tea utensils used for brewing significantly impacts the sensory and chemical attributes of tea. In order to assess the influence of various tea sets on the flavor and chemical composition of different tea varieties, a combination of sensory evaluation and high-performance liquid chromatography was employed. The results showed that the content of amino acids in the tea liquid brewed with tin tea utensils was relatively higher, which could exhibit freshness in taste, thus suitable for brewing green tea and white tea. The content of polyphenols, soluble carbohydrates, and water extract in the tea liquid brewed with a porcelain tea set was relatively higher; the sweetness and thickness of the tea liquid were increased, so it was more beneficial to brew black tea. The purple sand tea set was suitable for brewing oolong tea and dark tea, and could endow their respective quality characteristics. Ultimately, these research findings provide a scientific basis for the selection of tea utensils tailored to different types of tea.

## 1. Introduction

There are a variety of tea types in China. The most common classification method is to divide Chinese tea into six categories according to different processing techniques and fermentation levels, namely green tea, yellow tea, dark tea, white tea, oolong tea, and black tea. The first step in green tea processing is to destroy enzymes at high temperatures, which is also the key step. Green tea is a non-fermented tea. The processing of yellow tea is to add a process of yellowing [1,2] on the basis of green tea. Yellow tea is a lightly fermented tea. The processing of dark tea involves adding a process of pile fermenting on the basis of green tea and ferment the tea through microorganisms [3]. Dark tea is a post-fermented tea. The processing of white tea, which is a micro-fermented tea, mainly uses sunlight for withering [4]. The key step in the processing of oolong tea is the rotation course (Zuo-Qing), which controls the degree of fragmentation of tea cells in order to achieve the purpose of controlling the degree of fermentation. Oolong tea is a semi-fermented tea [5]. Black tea is a fully fermented tea. Through withering, rolling, and fermentation, the polyphenols in tea are oxidized as much as possible into the substances of theaflavins and thearubigins, thus forming its special flavor.

Each type of tea has its own unique flavor, and the formation of quality is closely related to the variety of the tea plant [6], the environmental conditions of the tea plant [7], the cutting, picking, shading, and other cultivation techniques [8], and the processing technology [9,10]. Brewing is also a key step that determines the flavor; different brewing methods will give the tea different flavors. Water quality is another factor; different pH values, the total dissolved solids (TDS) content, and the ions contained will affect the volatile and non-volatile components [11,12,13]. It is necessary to choose reasonable brewing parameters according to the characteristics of different teas [14]. It is generally believed that green tea can be brewed at about 85 °C, and it is brewed no more than three times; the brewing time needs to be adjusted according to the shape of the tea [15,16,17,18]. Oolong tea is characterized by multiple brewing times and a rich flavor and is generally brewed at about 95 °C [19,20]. When brewing black tea, the concentrations of the water extract, theaflavins, and thearubigins are generally used as the evaluation criteria for the appropriate concentration. It is appropriate to brew black tea two times to keep a suitable taste, and the selection of the tea-to-water ratio may be more important [21,22]. For white tea, the main characteristic is “fresh”, so it can be steeped in cold water for a long time [23]. However, hot water could significantly increase the extraction rate of tea polyphenols and amino acids [24]. Temperature has the greatest influence on the concentration of intrinsic components in the tea extract, and the most suitable temperature for white tea is 100 °C [25,26]. Dark tea is a unique tea in China that uses mature raw materials. Due to the low leaching rate of its internal components, the brewing temperature is generally 100 °C, and the brewing time is generally over 5 min. In order to obtain a better taste, the tea-to-water ratio should not be greater than 1:75 [3,27].

In addition to water and brewing methods, tea sets also play an important role. Tea was born by water and nurtured by utensils, and the latter are always combined with and influenced by the development of tea culture, which gives it various features and aesthetic characteristics [28]. Throughout the history of Chinese tea utensils, their textures have mainly included pottery, metal, porcelain, bamboo, glass, purple sand, lacquerware, and enamel, each influencing different aspects of tea infusion [29,30]. However, there are few studies reporting the influence of different tea utensils on the brewing of different teas. The purpose of this article is to explore the infusion characteristics of different teas when brewing with different tea sets and provide a theory for scientifically selecting tea sets for brewing different teas.

## 2. Results and Discussion

### 2.1. Sensory Evaluation of Teas Brewed with Various Tea Sets

**Figure 1:**
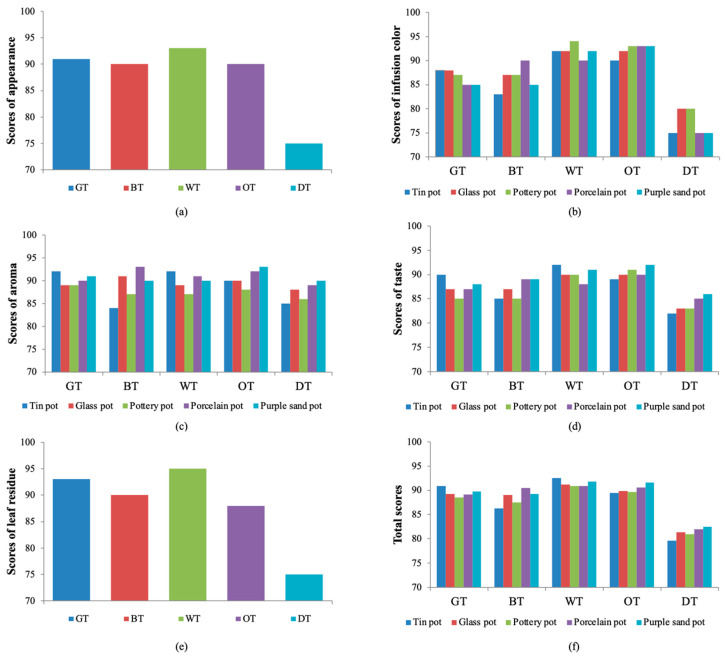
The scores of sensory evaluation. The scores of (**a**) appearance, (**b**) infusion color, (**c**) aroma, (**d**) taste, (**e**) leaf residue, and (**f**) total scores of different teas brewed in different tea sets. GT, BT, WT, OT, and DT represent green tea, black tea, white tea, oolong tea, and dark tea, respectively.

**Figure 2:**
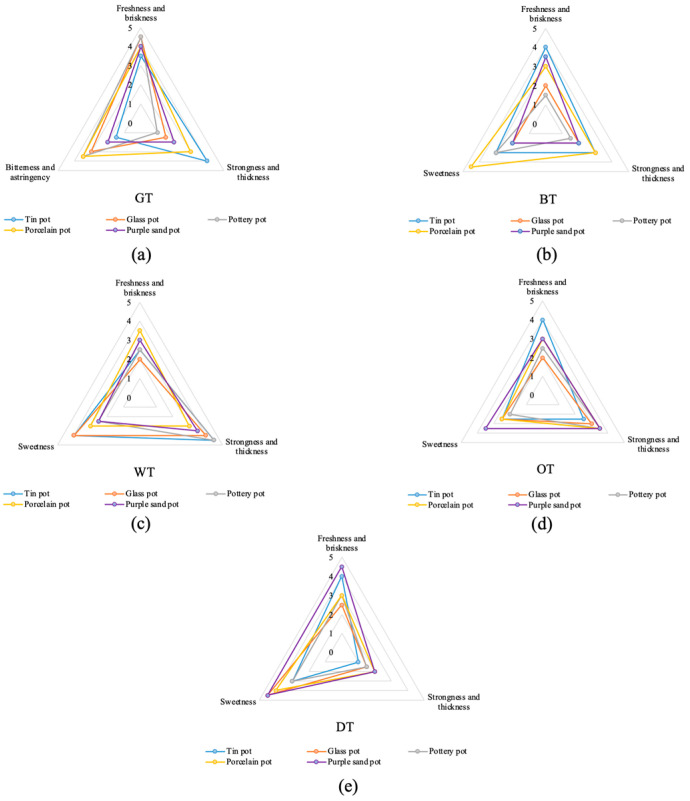
Taste profiles of tea liquor. (**a**) Green tea, (**b**) black tea, (**c**) white tea, (**d**) oolong tea, and (**e**) dark tea brewed in different tea sets.

In this experiment, sensory evaluations of five representative teas were carried out using tea sets with different textures (Table 1). It was found that when the same tea was brewed with different tea sets, it had different characteristics, including color, aroma, and taste. From the comprehensive score, green tea (90.85 points) and white tea (92.55 points) presented in a tin set had a better flavor. Porcelain improved the quality of black tea (90.45 points) (Figure 1). The purple sand tea set gave oolong tea (91.55 points) and black tea (82.5 points) a better flavor. After analyzing the different properties of tea soup (Figure 2), we found that brewing green tea with a tin tea set produced the best freshness and the lowest bitterness. Brewing black tea with the porcelain tea set produced the best sweetness and freshness. Brewing white tea with the tin tea set and glass tea set gave good performance in terms of freshness and sweetness, while its thickness performed badly in the glass tea set. The purple sand tea set can improve the sweetness and freshness in oolong tea and dark tea, especially for the latter, which is also the best in terms of thickness.

**Figure 3:**
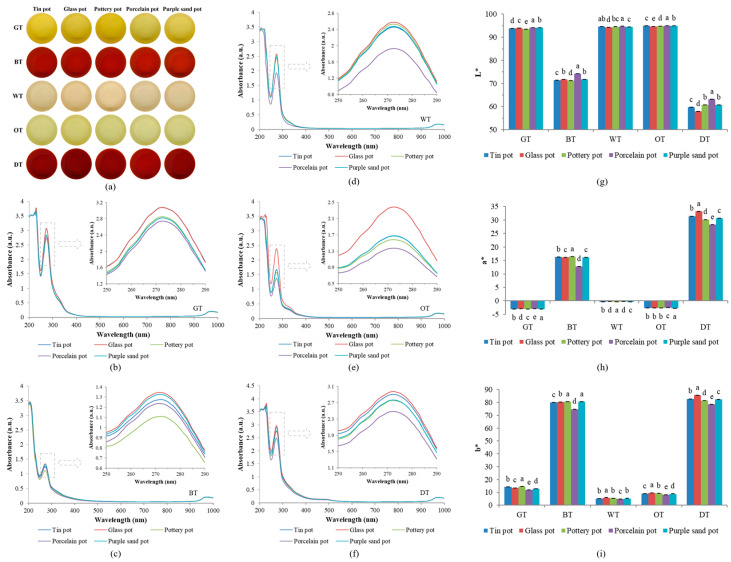
Color profiles of tea infusion. (**a**) Photographs of tea infusion from different teas brewed in different tea sets. (**b**–**f**) UV–vis spectra of (**b**) green tea, (**c**) black tea, (**d**) white tea, (**e**) oolong tea, and (**f**) dark tea brewed in different tea sets. (**g**–**i**) The values of (**g**) brightness, (**h**) red–green hue, and (**i**) yellow–blue hue of different teas brewed in different tea sets. Note: the lowercase letters represent the significant differences between different treatments.

Variations in color were observed when infusing the same teas utilizing different tea sets (Figure 3a). UV-vis spectrums of the tea infusion show an evident absorption peak only at 273 nm (Figure 3b–f), which is consistent with the characteristic absorption peak of tea polyphenols [31]. This suggests that the tea infusion contained substantial tea polyphenols, whose contents were different between teas brewed in various tea sets. Further, the brightness (L*), red-green hue (a*), and yellow-blue hue (b*) of the infusions were measured using a colorimeter to quantitatively analyze the colors. The brightness of the tea infusions brewed in the porcelain tea set was higher, while the glass and pottery sets caused darker tea infusions (Figure 3g). As for the red-green hue, the tea infusions brewed in porcelain sets were grayer, which meant less red or less green (Figure 3h). Brewing using glass and pottery sets made the tea infusions yellower (Figure 3i).

### 2.2. Effect of Different Tea Sets on Chemical Compounds and Sensory Attributes of Various Teas

**Figure 4:**
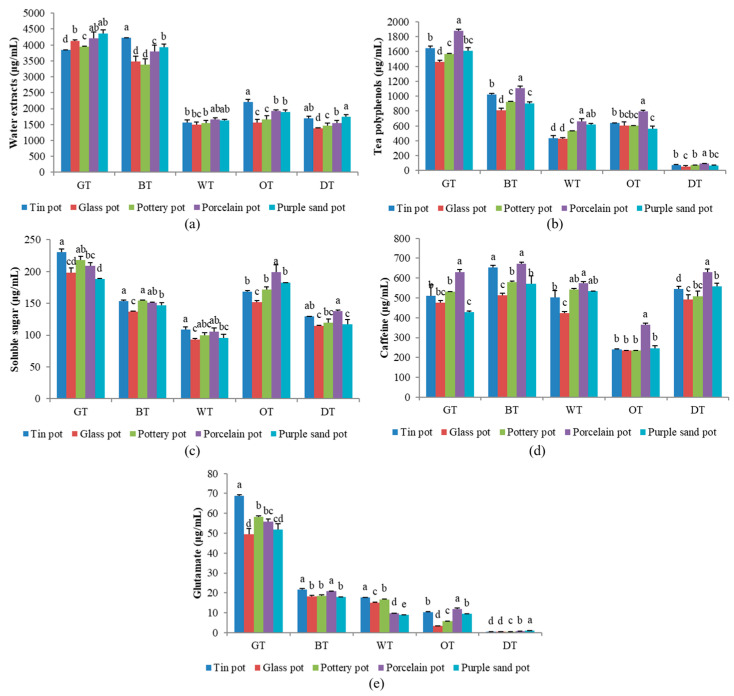
The contents of flavor compounds. The contents of (**a**) water extract, (**b**) tea polyphenols, (**c**) soluble sugar, (**d**) caffeine, and (**e**) glutamate of different tea infusions brewed in different tea sets. GT, BT, WT, OT, and DT represent green tea, black tea, white tea, oolong tea, and dark tea, respectively. Note: the lowercase letters represent the significant differences between different treatments.

A comprehensive analysis was conducted on a total of 19 chemical compounds in all five types of tea samples brewed using five distinct tea sets. These compounds encompassed five catechins, four flavanol glycosides, caffeine, six amino acids, the total amino acid content, soluble carbohydrates, and aqueous extract. The concentrations of these components are shown in Tables S1 and S2. Moreover, Figure 4 elucidates the significant variations in the chemical contents of the different tea set treatments. The sensory attributes associated with these compounds are documented in Table 2.

Principal component analysis (PCA) was used to extract the characteristic features of sensory attributes and chemical compounds in different teas. All the five types of tea (green tea, black tea, white tea, oolong tea, and dark tea) were analyzed by PCA, respectively. For each type of tea, a total of twenty-two parameters, including five sensory attributes and nineteen chemical compounds, of the tea samples brewed with the five tea sets were analyzed. All the scores of sensory attributes and concentrations of the chemical compounds acted as variables, and their features were extracted by reducing the dimensionality using PCA.

#### 2.2.1. Green Tea

**Figure 5:**
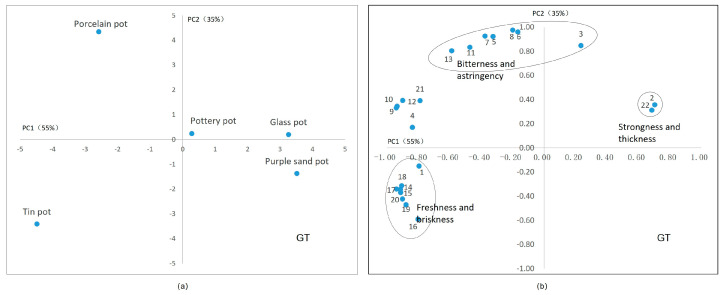
Principal component analysis of green tea’s taste. (**a**) The scores of tea sets in two main components. (**b**) The correlation between the chemical components in tea infusion and the sensory factors. Note: 1. umami, 2. strong, 3. astringency, 4. gallic acid (GA), 5. epigallocatechin gallate (EGCG), 6. gallocatechin gallate (GCG), 7. epicatechin gallate (ECG), 8. catechin gallate (CG), 9. myricetin glycosides, 10. kaempferide glycosides, 11. quercetin glycosides, 12. vitexin glycosides, 13. caffeine (CAF), 14. aspartic acid (Asp), 15. glutamate (Glu), 16. asparagine (Asn), 17. glutamine (Gln), 18. γ–aminobutyric acid (γ–GABA), 19. theanine (Thea), 20. amino acid, 21. soluble sugar, 22. water extract. Note: the oval in subfigure (**b**) represent these attributes in circles have similar characteristics.

The relationship between sensory attributes and chemical compounds in green tea is exhibited in Figure 5. The first two principal components made up 90% of variance with PC1 = 55% and PC2 = 35%. Figure 4a shows the scores of the tea sets in the first two components. Figure 5b shows a variable correlation map, providing the relationships between sensory attributes and chemical compounds. In Figure 5a, teas brewed with a glass pot and purple sand pot show the same values in PC1 (on the horizontal axis). Compared to Figure 5b, the stongness and thickness of the aqueous extract were high positively correlated with PC1. This indicates that the teas brewed with the glass pot and purple sand pot exhibited a strong and thick taste. Teas brewed using the porcelain and tin sets exhibited opposite values in PC2. The positive values in PC2 correspond to bitterness and astringency, which are located close to ester-type catechins, quercetin glycosides, and caffeine. The negative values in PC2 correspond to freshness and briskness and seven amino acids. The results showed that green tea brewed with the porcelain set was more likely to exhibit bitter and astringency characteristics with high concentrations of catechins, while green tea exhibited a fresh and brisk taste when brewed in the tin set, with high concentrations of amino acids.

#### 2.2.2. Black Tea

**Figure 6:**
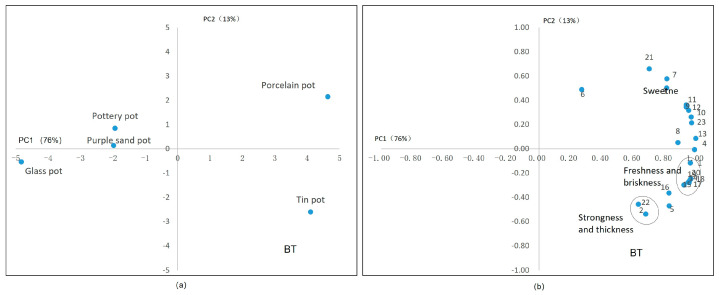
Principal component analysis of black tea’s taste. (**a**) The scores of tea sets in two main components. (**b**) The correlation between the chemical components in tea infusion and the sensory factors. Note: 1. umami, 2. strong, 3. astringency, 4. GA, 5. EGCG, 6. GCG, 7. ECG, 8. CG, 9. myricetin glycosides, 10. kaempferide glycosides, 11. quercetin glycosides, 12. vitexin glycosides, 13. CAF, 14. Asp, 15. Glu, 16. Asn, 17. Gln, 18. γ–GABA, 19. Thea, 20. amino acid, 21. soluble sugar, 22. water extract. Note: the oval in subfigure (**b**) represent these attributes in circles have similar characteristics.

In the PCA results for black tea, the first two principal components had 89% of variance: PC1 = 76% and PC2 = 13%. The PC1 values could reveal most characteristics of black teas treated with different tea sets. In Figure 6a, the porcelain tea set and tin tea set show opposite positions to the other tea sets on the horizontal axis, especially to the glass pot. In Figure 6b, the porcelain tea set is correlated with sweetness, while the tin tea set is related to a strong and fresh taste. The other tea sets led to a light taste and low concentrations of chemicals. As a result of the fermentation process, some catechins in black tea are oxidized, so there is no significant correlation between ester catechins and taste (Figure 6b), while the content of tea polyphenols is positively correlated with flavonoids and caffeine content, similar with Scharbert’s results [37], which identified that flavonol-3-glycosides contribute to astringency and increase the bitterness of caffeine in tea infusions. The content of soluble sugar is closely linked to the perception of sweetness, and the thickness is positively correlated with the content of water extract.

#### 2.2.3. White Tea

**Figure 7:**
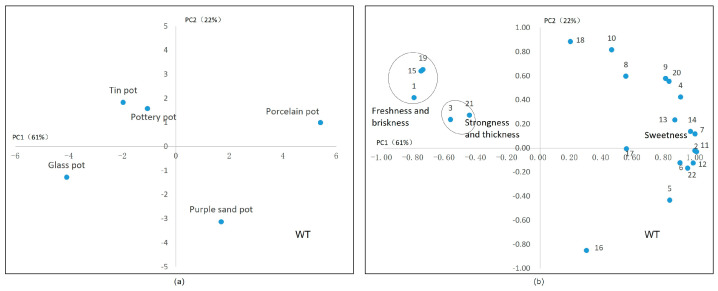
Principal component analysis of white tea’s taste. (**a**) The scores of tea sets in two main components. (**b**) The correlation between the chemical components in tea infusion and the sensory factors. Note: 1. umami, 2. strong, 3. astringency, 4. GA, 5. EGCG, 6. GCG, 7. ECG, 8. CG, 9. myricetin glycosides, 10. kaempferide glycosides, 11. quercetin glycosides, 12. vitexin glycosides, 13. CAF, 14. Asp, 15. Glu, 16. Asn, 17. Gln, 18. γ–GABA, 19. Thea, 20. amino acid, 21. soluble sugar, 22. water extract. Note: the oval in subfigure (**b**) represent these attributes in circles have similar characteristics.

For white tea, the variances of PC1 (61%) and PC2 (22%) were also high and could explain the characteristics of white teas treated with different tea sets. As shown in Figure 7a, teas brewed with a glass pot and purple sand pot show similar values in PC1 (on the horizontal axis). It was found that the freshness was closely related to the content of glutamic acid and theanine (Figure 7b). As for the sweetness, it is significantly positively correlated with soluble sugar, the total amount of amino acids, and the content of aspartic acid, as well as the content of caffeine and flavonoids. The integrated analysis of Figure 7a,b reveals a positive correlation between the tin tea sets and freshness, and demonstrates that both the tin and ceramic tea sets exhibited superior thickness performance. This result is similar to those of Chen Yi [38], Liu Panpan [34], and Liu Shuang [39], whose research indicated that the sweetness and freshness in Baihao Yinzhen was mainly contributed by aspartic acid and glutamate, respectively.

#### 2.2.4. Oolong Tea

**Figure 8:**
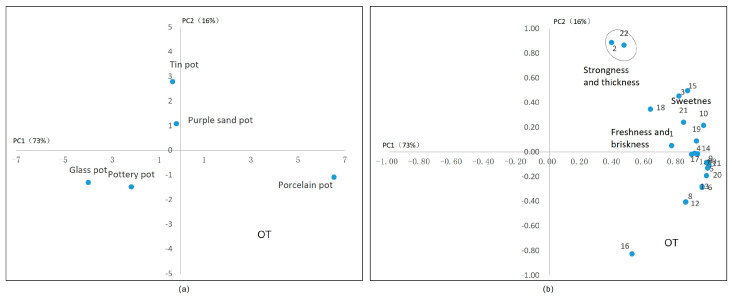
Principal component analysis of oolong tea’s taste. (**a**) The scores of tea sets in two main components. (**b**) The correlation between the chemical components in tea infusion and the sensory factors. Note: 1. umami, 2. strong, 3. astringency, 4. GA, 5. EGCG, 6. GCG, 7. ECG, 8. CG, 9. myricetin glycosides, 10. kaempferide glycosides, 11. quercetin glycosides, 12. vitexin glycosides, 13. CAF, 14. Asp, 15. Glu, 16. Asn, 17. Gln, 18. γ–GABA, 19. Thea, 20. amino acid, 21. soluble sugar, 22. water extract. Note: the oval in subfigure (**b**) represent these attributes in circles have similar characteristics.

According to the analysis of the components and taste of oolong tea (Figure 8), we found that it is hard to distinguish sweetness and freshness, which are positively related to glutamic acid, theanine, γ-aminobutyric acid, and soluble sugar. The contents of ester catechins, caffeine, and flavonoids exhibit a positive correlation (PC1). Therefore, using a porcelain tea set to brew oolong tea will introduce astringency, thus lowering the quality of the tea. The purple sand tea set and tin tea set are positively correlated with fresh and sweet substances (PC2) and have a certain negative correlation with bitter substances. Therefore, the tea has a better taste. Moreover, sensory evaluation found that the purple sand tea set could greatly improve the aroma, perhaps due to its porosity and permeability.

#### 2.2.5. Dark Tea

**Figure 9:**
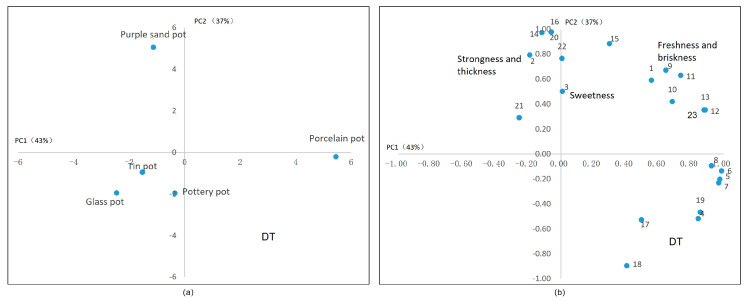
Principal component analysis of dark tea’s taste. (**a**) The scores of tea sets in two main components. (**b**) The correlation between the chemical components in tea infusion and the sensory factors. Note: 1. umami, 2. strong, 3. astringency, 4. GA, 5. EGCG, 6. GCG, 7. ECG, 8. CG, 9. myricetin glycosides, 10. kaempferide glycosides, 11. quercetin glycosides, 12. vitexin glycosides, 13. CAF, 14. Asp, 15. Glu, 16. Asn, 17. Gln, 18. γ–GABA, 19. Thea, 20. amino acid, 21. soluble sugar, 22. water extract.

After analyzing the flavoring substances in dark tea (Figure 9), we found that the thickness was positively correlated with the water extract, total amino acids, and some amino acid components, as well as with the glycan and soluble sugar content (PC2). This observation aligns with the taste attributes commonly associated with dark tea. However, the total amount of amino acids in dark tea is relatively low, and it is difficult to perform a cluster analysis between them, so its freshness is hard to capture. The total amount of tea polyphenols is positively correlated with flavonoids, and can be clustered, but has a weak correlation with catechins (PC2), which is related to the pile process. A purple sand tea set can introduce a stale flavor as well as sweetness and mellowness to dark tea (PC2), so it is more suitable for brewing dark tea.

### 2.3. Matching of Different Tea Sets and Characteristic Taste Substances

The results of the principal component analysis revealed that the utilization of tin tea utensils for brewing tea significantly elevated the levels of glutamic acid and theanine, consequently enhancing the freshness of the tea infusion. Using a porcelain tea set to brew tea can increase the soluble sugar, flavonoid content, and total amount of tea polyphenols, so it can increase the concentration and sweetness of the tea infusion. When using a glass tea set to brew tea, the total amount of tea polyphenols, the total amino acids in the tea soup, and the soluble sugar are lower due to the heat preservation of the tea set. Xu [40] found that tea sets with different thermal insulation properties have a greater impact on the dissolution of tea components, while the insulation of tea sets is closely related to the material. Metal has good heat conduction, so when using a tin pot, the temperature drops more rapidly. This is because metal has more free electrons. These free electrons can rapidly transfer heat in the metal lattice, enabling good heat conduction [41]. Pottery tea sets and purple sand tea sets have a relatively high void ratio [30,41] and the gas permeability and the adsorptivity are relatively good, so it is easier to adsorb small molecular groups in tea infusions, thereby improving the taste of the tea soup. The air permeability of a tea set refers to the characteristics of its voids or microchannels, which allow air to circulate freely between the interior and exterior of the tea set. The bidirectional exchange of air is considered an important contributor to enhancing the taste of the tea infusion. As an illustration, the double stomatal structure of a purple sand tea set allows for enough air exposure, stimulating the liberation of aromatic compounds from the tea leaves and facilitating the interaction of bitter and sour compounds with the air. Consequently, the tea acquires a more fragrant aroma, while the tea infusion becomes more mellow.

This study investigated the effect of different tea utensils on the sensory and chemical quality of different teas. Nevertheless, there are several limitations to be acknowledged in exploring the mechanisms of thermal properties, air permeability, and adsorptivity of tea utensils. Both the material composition and capacity of the utensil influence its heat dissipation and retention capabilities, which alter the rate and concentration of tea chemicals released, consequently leading to variations in the sensory quality of the tea. Therefore, the selection of an appropriate utensil is of importance when aiming to brew tea of superior quality. Further investigations will utilize quantitative tools and methodologies to explore these aspects in greater detail. For instance, thermal probes will be employed to monitor the time-dependent variations in solvent temperature, allowing for a comprehensive assessment of the heat exchange properties exhibited by various tea sets. Moreover, an optical profilometer will be utilized to precisely measure the shape and surface area of containers, enabling an in-depth analysis of the influence of heat loss rate on the quality of the brewed tea infusion. Additionally, an essential aspect of future research will focus on determining the adsorption capabilities of porous containers and the mechanism underlying gas exchange. To address these aspects comprehensively, the construction of pure standard solutions will be undertaken to systematically evaluate the impact of gas permeability and adsorption phenomena on the quality of the tea.

## 3. Materials and Methods

### 3.1. Tea Samples, Tea Sets, and Water

There were five types of tea products used in this study, which were purchased from commercial tea companies. They were green tea (West Lake Longjing, Gongpai), black tea (Dianhong Gongfu, Fengpai), white tea (Baihao Yinzhen, Pinpinxiang), oolong tea (Tieguanyin, Xuandetang), and dark tea (Liupao Tea, Wuzhou Zhongcha). All the tea samples had typical flavors.

The tea sets, with equal volume, included a tin tea set, glass tea set, pottery tea set, porcelain tea set, and purple sand tea set. A tin tea set is a vessel constructed by tin, a material characterized by its rigidity, flexibility, excellent sealing properties, and notable ductility. It is commonplace for a minor proportion of other metallic elements to be incorporated into the tin to enhance its hardness. In this study, the tin tea set was made of 97% tin (Sn), with an additional 3% copper (Cu) and antimony (Sb). Glass tea sets are made of glass. Glass has a hard, brittle, and transparent texture, and its main components are silica, calcium oxide, and sodium oxide. It is formed by mixing quartz sand, limestone, soda ash, etc., and melting, forming, and cooling at high temperatures. Pottery tea sets are utensils made of clay. Due to the different percentages of various metal oxides contained in clay and the differences in firing temperature, it can appear in different colors such as red, brown-black, white, gray, blue, and yellow. The firing temperature is generally around 1000 °C, and the ceramic texture is rough and loose. This experiment used a brown glazed pottery tea set. Porcelain tea sets are utensils made of feldspar, kaolin, and quartz as raw materials. They are made by mixing the raw materials, processing it into a dry shape, and firing it at a high temperature of around 1300 °C. Porcelain is generally glazed and has a hard and dense texture, a smooth surface, and low water absorption. Purple sand tea sets are utensils made of Yixing purple clay. The purple clay has a purple-red color, a delicate texture, strong plasticity, and good permeability. After forming, it is fired at a high temperature of 1150 °C. The mineral composition of purple mud belongs to the iron-containing clay–quartz–mica system, forming a uniform particle structure. The water utilized for brewing was obtained from Nongfu Spring Co. Ltd. (Hangzhou, China). Sourced from a natural deep lake, it is widely employed in the sensory evaluation of tea. The element concentrations of the water were as follows: Ca ≥ 4.00 μg/mL, Mg ≥ 0.50 μg/mL, K ≥ 0.35 μg/mL, Na ≥ 0.80 μg/mL, and metasilicic acid (H_2_SiO_3_) ≥ 1.80 μg/mL. The pH of the water was maintained at 7.3 ± 0.5.

### 3.2. Chemical Materials

Acetonitrile, methanol, and acetic acid were chromatographically pure and purchased from Tedia, Fairfield, OH, USA; o-phthalaldehyde (OPA), methyl chloroformate (FMOC), gallic acid, 8 catechin monomers, 3 alkaloid monomers, 16 kinds of flavonoids, and 20 kinds of amino acid standards were purchased from Shanghai Yuanye Biotechnology Co., Ltd. (Shanghai, China); and potassium dihydrogen phosphate, disodium hydrogen phosphate, sodium potassium tartrate, ferrous sulfate, and anthrone were analytically pure and purchased from Sinopharm Chemical Reagent Co., Ltd. (Shanghai, China).

### 3.3. Preparation of Tea Infusions

The five tea samples were brewed with different tea sets at a tea-to-water ratio of 1:50. When brewing, the teapot was filled with the same volume of water at a temperature of 100 °C. The infusion was filtered 5 min later, except West Lake Longjing tea, which was extracted within 4 min.

### 3.4. Analysis of Chemical Compounds

The water extract of tea soup was determined by referring to “Determination of Tea Leachate” (GB/T8305-2013) [42]; the tea polyphenol content was determined by referring to “Detection method of tea polyphenols and catechins in tea” (GB/T8313-2018) [43]. The total amount of free amino acids was determined by referring to “Measurement of Total Amount of Free Amino Acids in Tea” (GB/T8314-2013) [44]; the content of soluble sugars was determined by the anthrone sulfuric acid method.

Determination of catechins and alkaloids: HPLC-UV detection, column: Agilent TC-C18 column (4.6 mm × 250 mm, 5 μm); mobile phase: flow A was 3% acetonitrile +5% acetic acid; mobile phase B was 30% acetonitrile + 5% acetic acid; flow rate: 1 mL/min; elution procedure: 0 min B phase was 20%; at 0–40 min, it linearly rose to 75%, then immediately dropped to 20% for 5 min, and ended at 45 min; column temperature: 28 °C; detection wavelength: 280 nm; sample size: 10 μL. Determination of flavonoid content: HPLC-UV detection, column: Agilent TC-C18 column (4.6 mm × 250 mm, 5 μm). Detection of flavonoid content: mobile phase: flow A was 0.1% formic acid, mobile phase B was 0.1% acetonitrile, flow rate was 1 mL/min; elution procedure: 0 min B phase was 20%; at 0–40 min, it linearly rose to 40%, then immediately dropped to 20% for 5 min, and ended at 45 min; column temperature: 28 °C; detection wavelength: 360 nm; injection volume: 10 μL. Detection of free amino acid component content [45]: pre-column derivatization HPLC-FLD assay, column: Zorbax Eclipse-AAA column (4.6 mm × 150 mm, 3.5 μm). Mobile phase A was 40 mmol/L Na_2_HPO_3_, mobile phase B was acetonitrile/methanol/water = 45:45:10, flow rate: 1.5 mL/min; elution procedure: 0–18 min B phase increased linearly from 5% to 60%; at 18–23 min, it linearly rose to 100%, immediately dropped to 5% at 23 min and remained for 5 min, ending at 28 min; column temperature: 40 °C; excitation wavelength: 340 nm; emission wavelength: 450 nm; injection volume: 10 μL.

### 3.5. Sensory Analysis

According to the national standard of China Methods for Tea Sensory Evaluation (GB/T23776-2018) [46], the appearance, infusion color, aroma, taste, and leaf of each tea sample are, respectively, described with terms and scored. (The Methods for Tea Sensory Evaluation (GB/T23776-2018) can be found at the following link: https://std.samr.gov.cn/gb/search/gbDetailed?id=71F772D82CD3D3A7E05397BE0A0AB82A (Accessed on 21 February 2024)) The appearance was assessed in terms of dry leaf shape, maturity, color, and uniformity. The tea infusion color was scored according to brightness and clarity. The aroma was inspected according to flavor, strength, purity, and permanency. The taste was assessed in terms of freshness, strongness, astringency, and sweetness. Infused leaves were scored according to tea leaf maturity and color.

The total score was calculated according to Equation (1).

(1) Y=A×a+B×b+C×c+D×d+E×e

where Y is the total score; A,B,C,D,E  are the scores of appearance, infusion color, aroma, taste, and leaf residue, respectively; and a,b,c,d,e  are the weights of each factor which are shown in Table S3.

The taste profiles of the tea infusions were determined according to the national standard of China Sensory Analysis Method—Flavor Profile Test (GB 12313-90) [47], corresponding to the international standard Sensory Analysis—Methodology Flavor Profile Methods (ISO 6564-1985) [48]. The taste of the green tea infusion was divided into three sub-attributes, which were freshness and briskness, strongness and thickness, and bitterness and astringency. The taste profiles of the other teas (black tea, white tea, oolong tea, and dark tea) were evaluated based on freshness and briskness, strongness and thickness, and sweetness. The 5-point system with a precision of 0.5 was applied. Higher scores represented enhanced properties. The sensory evaluations were conducted by a panel of five experienced assessors, comprising three females and two males. Each assessor had professional training, with a cumulative experience of over 8000 h in the sensory analysis of tea. The sensory evaluations were conducted in a double-blind manner, ensuring that all panelists were unaware of any information pertaining to the teas. The outcomes and scores derived from the sensory assessment represent the consensus achieved by the panel following thorough deliberation.

### 3.6. Quantitative Analysis of Tea Infusion Color

Ultraviolet and visible spectrum (UV-vis) analysis was performed using a micro-plate reader (BioTek, Winooski, VT, USA). And the EGCG solution was placed in a 96-well quartz plate to measure its optical properties between 200 and 1000 nm. A spectrocolorimeter (CM-3600d, Konica Minolta, Hong Kong, China) was used to measure the brightness (L*), red-green hue (a*), and yellow-blue hue (b*) of the infusion.

### 3.7. Statistical Analysis

All the chemical compounds were analyzed in triplicate. Data processing was performed in Excel, with the data presented as means ± standard deviations. The differences between tea-brewing sets’ treatments were analyzed by Duncan’s new multiple range test in IBM SPSS Statistics 21 software (IBM, Armonk, NY, USA). *p* < 0.05 was considered as a significant difference. Correlations between variables and observations were analyzed using principal component analysis performed on XLSTAT 2018 (Addinsoft, New York, NY, USA).

## 4. Conclusions

Incorporating chemical analysis and sensory evaluation, this study determined that various types of teas have their respective optimal tea set pairings. Tin tea sets demonstrate optimal compatibility with green tea and white tea, effectively accentuating their freshness and briskness. Additionally, porcelain tea sets are deemed most suitable for brewing black tea, resulting in enhanced sweetness and richness. Moreover, the purple sand tea set is identified as the ideal choice for oolong tea and dark tea, effectively accentuating their unique stylistic characteristics. This study establishes a theoretical foundation for scientifically selecting appropriate tea utensils to brew different types of tea.

## Figures and Tables

**Table 1:** Sensory evaluation results of different teas brewed in different tea sets.

Samples	Utensils	Appearance	Infusion Color	Aroma	Taste	Leaf Residue
Green tea	Tin pot	Flat and straight, relatively smooth and flat, relatively even, relatively soft green, and relatively lustrous.	Apricot yellow, relatively bright.	Strong and sharp, relatively fragrant, showing a soft flavor.	Relatively strong and mellow, brisk, slightly bitter.	Fine and soft with many buds, relatively flowery, relatively even, approaching soft bright green.
	Glass pot		Apricot yellow, relatively bright.	Approaching strong, slightly dull odor, sufficient smoky aroma.	Strong and mellow, relatively brisk, little bitterness.	
	Pottery pot		Yellow and bright.	Higher briskness, soft flavor, slightly dull odor.	Strong and mellow, approaching brisk, little bitterness and astringency.	
	Porcelain pot		Quite deep yellow, relatively bright.	Higher briskness, relatively fragrant, showing a soft flavor.	Relatively strong and mellow, relatively fresh, little bitterness and astringency.	
	Purple sand pot		Quite deep yellow, relatively bright.	Strong and brisk, fragrant.	Approaching strong and mellow, approaching brisk, slightly bitter.	
Black tea	Tin pot	Bold, especially showing a golden pekoe-like appearance, relatively even, black bloom.	Red and relatively bright, little dullness.	Relatively stale flavor, slightly tainted aroma, approaching sweet, relatively dull odor.	Strong and approaching brisk, slight astringency, slightly sour.	Relatively soft and thick with many buds, red and bright.
	Glass pot		Red and relatively bright.	Highly sweet with a sweet fruity flavor.	Relatively mellow and thick, relatively sweet, slightly astringent.	
	Pottery pot		Quite deep red, relatively bright.	Approaching strong, slightly dull odor.	Approaching strong, slightly dull odor, slightly astringent.	
	Porcelain pot		Quite deep red, bright.	High sweetness, relatively fragrant.	Relatively strong and mellow, relatively sweet and brisk, slightly astringent, slightly sour.	
	Purple sand pot		Deep red, approaching brightness.	Relatively high sweetness, slightly dull odor.	Strong and relatively thick, slightly astringent.	
White tea	Tin pot	All buds are relatively fat and bold, covered by pekoe, silvery green.	Yellow and bright.	Sweet aroma and pekoe flavor.	Mellow and thick, sweet and fresh.	All buds are fat and thick, silvery green.
	Glass pot		Yellow and bright.	Relatively sweet (thin aroma), with a pekoe flavor.	Mellow and thick, sweet and fresh (slightly astringent).	
	Pottery pot		Apricot yellow and bright.	Slightly dull odor with sweet aroma.	Mellow and thick, relatively sweet and fresh, slightly dull odor.	
	Porcelain pot		Yellow little deep approaching bright.	Relatively sweet, showing a soft pekoe flavor.	Approaching strong and mellow, little astringency.	
	Purple sand pot		Yellow and bright.	Relatively sweet, showing a pekoe flavor.	Mellow and thick, relatively sweet and brisk.	
Oolong tea	Tin pot	Twisted particle shape, almost tight and heavy, quite pekoe-like, relatively even, frog skin alike green with jade green, relatively blooming.	Relatively bright honey green.	Clean and relatively fresh, approaching fragrant, slightly dull odor.	Approaching strong and mellow, approaching fresh, slightly dull odor.	Relatively thick and soft, green, and bright.
	Glass pot		Honey green and bright.	Clean and fresh, approaching fragrant.	Relatively mellow and thick, clean, and fresh.	
	Pottery pot		Honey green and bright.	Relatively grassy odor, slightly dull odor.	Relatively mellow and thick, relatively clean and fresh, slightly sour, has a Tieguanyin flavor.	
	Porcelain pot		Honey green and bright.	Relatively fragrant, clean and fresh, has a Yin-like flavor.	Relatively mellow and thick, little astringency.	
	Purple sand pot		Honey green and bright.	Relatively fragrant, clean and fresh, showing a Yin-like flavor.	Mellow and thick, clean and fresh, showing a Yin-like flavor.	
Dark tea	Tin pot	Coarse and loose with many stems, auburn, and quite dry.	Auburnish red and dull.	Relatively pure.	Approaching strong, slightly coarse.	Slightly hard with many stems, black auburn, quite dull.
	Glass pot		Deep red, approaching bright.	Relatively pure, showing a stale flavor.	Mellow, slightly sweet with a stale flavor.	
	Pottery pot		Deep red, approaching bright.	Relatively pure, has a stale flavor and slightly dull odor.	Mellow, slightly sweet with a stale flavor.	
	Porcelain pot		Auburnish red and dull.	Pure, has a stale flavor.	Mellow, slightly sweet, stale flavor.	
	Purple sand pot		Auburnish red and dull.	Pure, showing an aged flavor.	Relatively sweet and mellow, relatively thick, with stale flavor.	

**Table 2:** Sensory properties of flavor compounds.

Compounds	Sensory Description	Reference
Water extract	Strong	Chen, 2014 [32]
Tea polyphenols	Bitter taste	Tea review and inspection
Soluble sugar	Sweet taste	Tea review and inspection
Theobromine	Slightly bitter	Tea biochemistry
Tea base	Bitterness	Tea biochemistry
Caffeine	Bitterness	Tea review and inspection
Gallic acid	Sour taste	Tea review and inspection
Gallocatechin	Astringency	Tea review and inspection
Epigallocatechin	Astringency	Tea review and inspection
Catechin	Bitter taste	Tea review and inspection
Epicatechin	Slightly weak and sweet	Tea review and inspection
Epigallocatechin gallate	Strong bitter taste	Tea review and inspection
Gallocatechin gallate	Strong bitter taste	Tea review and inspection
Epicatechin gallate	Strong bitter taste	Tea review and inspection
Catechin gallate	Strong bitter taste	Tea review and inspection
Salicylone-3-O-galactoside	Bitterness	Tea biochemistry
Myricetin-3-O-glucoside	Bitterness	Tea biochemistry
Vitexin-2-O-rhamnoside	Bitterness	Tea biochemistry
Rutin	Bitterness	Tea biochemistry
Vitexin	Bitterness	Tea biochemistry
Myricetin-3-O-rhamnoside	Bitterness	Tea biochemistry
Tripletin 3-D-galactoside (hyperin)	Bitterness	Tea biochemistry
Phospholipidin 3-β-D glucose compound	Bitterness	Tea biochemistry
Kaempferol 3-O-β-rutinoside	Bitterness	Tea biochemistry
Kaempferol-glucoside	Bitterness	Tea biochemistry
Cytosine	Bitterness	Tea biochemistry
Myricetin	Bitterness	Tea biochemistry
Hesperidin	Bitterness	Tea biochemistry
Aspartic acid	Fresh, sweet, and sour	Tea review and inspection
Glutamate	Fresh, sweet, and sour	Tea review and inspection
Asparagine	Fresh, sweet, and sour	Tea review and inspection
Serine	Sweet taste	Tea review and inspection
Glutamine	Fresh, sweet, and sour	Tea review and inspection
Histidine	Bitterness	Peng, 2021 [33]
Glycine	Sweet taste	Tea review and inspection
Threonine	Sweet taste	Liu, 2014 [34]
Arginine	Bitterness	Peng, 2021 [33]
Alanine	Sweet taste	Tea review and inspection
γ-aminobutyric acid	Bitter taste inhibitor; <100 mg/L can inhibit the bitterness of caffeine	Cui, 2013 [35]
Theanine	Fresh and sweet	Tea review and inspection
Tyrosine	Bitterness	Liu, 2014 [34]
Valine	Bitterness	Liu, 2014 [34]
Methionine	Bitterness	Ye, 2015 [36]
Tryptophan	Bitterness	Peng, 2021 [33]
Phenylalanine	Bitterness	Liu, 2014 [34]
Isoleucine	Bitterness	Scharbert, 2005 [37]
Leucine	Bitterness	Liu, 2014 [34]
Lysine	Bitterness	Peng, 2021 [33]

## Data Availability

Data are contained within the article and Supplementary Materials.

## Supplementary Materials

The following supporting information can be downloaded at: https://www.mdpi.com/article/10.3390/plants13050669/s1, Table S1. Contents of water extract, tea polyphenols, soluble sugar, and CAF in tea infusions (μg/mL). Table S2. Contents of flavonoids and amino acids in tea infusions (μg/mL). Table S3. Weight of evaluation factors for each tea category (%).
