# Supplementary material for: Influence of Various Tea Utensils on Sensory and Chemical Quality of Different Teas
Source: Plants (Basel). 2024 Feb 28;13(5):669. doi: 10.3390/plants13050669 (PMC10933942; doi:10.3390/plants13050669)
Supplement: Supplementary file 1 [file plants-13-00669-s001.zip › plants-2861044-supplementary.pdf]

**Table S1.** Contents of water extract, tea polyphenols, soluble sugar, and CAF in tea infusions (μg/mL).

| tea | tea set | Water extract | Tea polyphenols | Soluble sugar | CAF | GA | EGCG | GCG | ECG | CG |
|---|---|---|---|---|---|---|---|---|---|---|
| green tea | Tin pot | 3838.21±5.00d | 1646.77±26.52b | 230.15±5.11a | 509.89±56.72b | 64.89±0.93a | 698.85±21.50bc | 47.06±1.70b | 363.74±6.36c | 11.48±2.23b |
| | Glass pot | 4133.40±26.74b | 1458.65±23.74d | 198.28±7.28cd | 474.97±12.28bc | 56.23±0.23b | 718.64±20.36bc | 63.52±14.53b | 371.69±22.27bc | 14.64±3.51b |
| | Pottery pot | 3936.57±31.58c | 1570.41±7.65c | 217.57±6.04ab | 530.95±0.45b | 56.27±6.02b | 902.03±139.84ab | 59.09±1.12b | 413.11±10.53b | 15.53±3.49b |
| | Porcelain pot | 4206.54±196.12ab | 1876.3±21.83a | 208.81±4.81bc | 630.65±12.74a | 60.77±3.38ab | 1102.79±124.27a | 110.68±20.87a | 523.63±21.22a | 25.46±4.37a |
| | Purple sand pot | 4362.42±103.62ab | 1607.22±44.51bc | 187.93±1.23d | 428.79±5.12c | 43.95±1.24a | 652.34±9.67c | 49.77±0.86b | 329.24±22.58c | 11.63±1.15b |
| black tea | Tin pot | 4220.00±5.70a | 1020.05±17.54b | 153.38±1.52a | 653.41±11.67a | 83.04±8.15a | 72.89±0.16a | 5.74±0.34c | 38.27±1.41b | 2.19±0.33a |
| | Glass pot | 3477.95±168.87d | 811.16±27.93d | 136.66±0.99c | 512.47±12.16c | 54.48±3.69c | 46.77±2.18d | 4.91±0.06c | 34.50±1.80c | 1.47±0.02b |
| | Pottery pot | 3377.30±187.01d | 923.10±10.04c | 153.96±1.25a | 578.84±4.62b | 68.83±3.95b | 56.63±2.84bc | 5.17±0.25c | 37.51±0.38bc | 1.96±0.09a |
| | Porcelain pot | 3795.73±194.97c | 1105.78±29.91a | 151.14±0.94ab | 673.15±6.97a | 82.79±0.55a | 59.10±0.14b | 13.03±0.07b | 45.32±0.47a | 2.18±0.01a |
| | Purple sand pot | 3922.76±109.30b | 904.51±17.56c | 146.77±3.90b | 571.65±40.65b | 64.68±3.65bc | 51.13±3.29cd | 14.88±1.53a | 37.57±1.95bc | 2.00±0.02a |
| white tea | Tin pot | 1560.80±79.58b | 434.04±35.21d | 108.83±4.13a | 501.61±35.46b | 38.74±1.16b | 181.19±18.34c | 6.80±1.00c | 128.34±14.93b | 4.14±0.15b |
| | Glass pot | 1493.50±90.14bc | 425.74±16.12d | 93.31±1.14c | 422.08±9.68c | 23.60±0.74c | 206.52±7.62c | 9.81±2.50bc | 120.05±15.92b | 4.11±0.30b |
| | Pottery pot | 1545.01±84.15b | 532.32±3.53c | 99.82±3.51abc | 542.49±5.42ab | 41.71±2.57b | 255.39±3.38b | 10.05±2.68bc | 135.32±4.83ab | 4.15±0.17b |
| | Porcelain pot | 1658.43±48.47ab | 664.64±32.24a | 105.66±5.27ab | 573.27±8.80a | 56.36±3.03a | 305.47±13.47a | 18.60±0.12a | 161.88±6.45a | 6.32±0.06a |
| | Purple sand pot | 1634.68±24.46ab | 619.09±12.76ab | 95.73±5.80bc | 533.86±0.36ab | 37.41±1.46b | 312.07±1.53a | 12.30±1.14b | 139.60±0.54ab | 2.80±0.01c |
| oolong tea | Tin pot | 2209.20±73.05a | 640.11±1.92b | 168.14±2.09b | 239.80±2.55b | 4.67±0.47b | 222.30±5.99b | 8.40±0.66c | 60.47±0.55bc | 3.62±0.08b |
| | Glass pot | 1560.76±101.32c | 606.88±44.12bc | 151.51±2.92c | 234.67±1.28b | 3.98±0.11c | 174.45±1.64c | 8.20±0.26bc | 50.36±0.14d | 3.63±0.01b |
| | Pottery pot | 1657.14±120.65c | 604.41±2.08bc | 171.31±4.14b | 234.91±0.70b | 4.10±0.13bc | 213.02±3.28b | 8.20±0.25c | 56.66±0.09c | 3.70±0.03b |
| | Porcelain pot | 1921.15±41.37b | 792.73±18.01a | 199.22±10.99a | 365.45±7.78a | 5.59±0.05a | 357.94±3.40a | 21.96±0.48a | 90.00±3.32a | 3.82±0.02a |
| | Purple sand pot | 1884.56±69.62b | 563.60±34.41c | 182.63±0.09b | 247.15±11.05b | 3.93±0.03c | 221.84±16.56b | 10.07±0.35b | 61.26±0.35b | 3.71±0.01a |
| dark tea | Tin pot | 1686.07±73.23ab | 71.71±9.73b | 129.34±0.32ab | 544.52±12.82d | 23.30±0.09b | 2.27±0.09c | 0.30±0.01c | 3.91±0.16c | 7.38±0.07b |
| | Glass pot | 1380.83±23.25d | 55.11±7.77c | 114.84±0.09c | 492.66±22.43c | 24.65±1.15c | 2.17±0.19cd | 0.22±0.01d | 3.16±0.18d | 6.14±0.19d |
| | Pottery pot | 1463.17±79.17c | 71.04±0.68b | 119.38±5.96bc | 508.99±25.37bc | 27.09±1.19d | 3.52±0.00b | 0.47±0.01b | 4.45±0.05b | 6.57±0.25c |
| | Porcelain pot | 1548.83±84.20b | 91.25±0.69a | 137.61±1.47a | 629.32±17.14a | 32.40±0.84a | 7.39±0.26a | 0.94±0.06a | 7.78±0.27a | 9.38±0.04a |
| | Purple sand pot | 1743.86±61.69a | 69.22±1.02bc | 116.6±7.52c | 557.89±15.44b | 20.21±0.13d | 1.86±0.00d | 0.27±0.02cd | 2.95±0.24d | 6.39±0.04cd |

**Table S2.** Contents of flavonoids and amino acids in tea infusions (μg/mL).

| tea | tea set | Myricetin-glycosides | Vitexin-glycosides | Quercetin-glycosides | Kaempferide-glycosides | Asp | Glu | Asn | Gln | γ-GABA | Thea |
|---|---|---|---|---|---|---|---|---|---|---|---|
| green tea | Tin pot | 6.79±0.29a | 4.47±0.16a | 1.05±0.15b | 0.84±0.08a | 48.98±0.78a | 68.85±0.50a | 8.67±0.27a | 53.54±0.54a | 7.93±0.62a | 347.77±1.22a |
| | Glass pot | 5.21±0.01bc | 3.72±0.10b | 0.24±0.00b | 0.65±0.06b | 35.50±2.54d | 49.47±2.82d | 5.53±0.05c | 32.84±1.37b | 5.80±0.07d | 238.85±4.77d |
| | Pottery pot | 5.60±0.03bc | 4.31±0.20a | 0.35±0.01b | 0.75±0.03ab | 42.19±1.21b | 58.30±0.32b | 6.03±0.13b | 38.32±1.13b | 7.02±0.20b | 276.28±11.71b |
| | Porcelain pot | 5.90±0.95ab | 4.62±0.28a | 0.35±0.01b | 0.84±0.07a | 39.94±0.47bc | 55.77±1.39bc | 5.77±0.04bc | 39.59±7.10b | 6.63±0.15bc | 263.29±0.85bc |
| | Purple sand pot | 4.44±0.02c | 3.54±0.13b | 17.37±5.36a | 0.61±0.01b | 36.75±0.74cd | 51.84±3.04cd | 5.78±0.04bc | 33.61±0.94b | 6.16±0.05cd | 250.58±3.45cd |
| black tea | Tin pot | 5.00±0.34b | 11.04±0.58b | 9.58±0.14b | 10.55±0.00b | 17.97±0.06a | 21.71±0.62a | 16.22±0.86a | 9.91±0.19a | 4.38±0.08a | 190.84±7.25a |
| | Glass pot | 3.85±0.16d | 8.76±0.24d | 7.43±0.29c | 8.20±0.28e | 15.32±0.18b | 18.27±0.52b | 14.37±0.24bc | 8.36±0.01d | 3.32±0.50c | 158.86±1.78b |
| | Pottery pot | 4.53±0.04bc | 10.10±0.52bc | 8.49±0.30bc | 9.48±0.11c | 15.94±0.60b | 18.39±0.57b | 14.58±0.81bc | 8.72±0.08c | 3.66±0.00bc | 160.26±6.69b |

| | | | | | | | | | | | |
|---|---|---|---|---|---|---|---|---|---|---|---|
| | Porcelain pot | 5.67±0.08a | 12.38±0.14a | 11.30±0.17a | 11.36±0.05a | 17.24±0.08a | 20.75±0.06a | 15.41±0.06ab | 9.51±0.01b | 4.12±0.03ab | 183.39± 1.82a |
| | Purple sand pot | 4.50±0.13c | 9.74±0.07c | 9.17±1.01b | 8.83±0.16d | 15.54±0.81b | 18.01±0.01b | 13.77±0.21c | 8.53±0.16cd | 3.70±0.22bc | 162.73±6.97b |
| white tea | Tin pot | 3.76±0.03c | 0.61±0.00d | 10.96±0.01c | 0.67±0.11ab | 16.26±0.44c | 17.69±0.01a | 23.04±1.43c | 10.71±0.02a | 12.95±0.11b | 236.28±2.79a |
| | Glass pot | 2.80±0.03d | 0.48±0.01e | 9.50±0.05d | 0.47±0.01c | 22.95±0.03ab | 15.18±0.01c | 19.90±0.06c | 7.82±0.03c | 12.20±0.06b | 226.80±0.47b |
| | Pottery pot | 4.08±0.14b | 0.70±0.01c | 11.83±1.04c | 0.65±0.01ab | 16.10±0.98c | 16.72±0.30b | 23.22±0.70d | 6.52±0.35d | 14.23±0.71a | 236.78±1.05a |
| | Porcelain pot | 5.17±0.10a | 2.40±0.01a | 20.73±0.43a | 0.68±0.01a | 22.08±0.49b | 9.82±0.04d | 38.91±0.52a | 10.99±0.14a | 15.06±0.06a | 174.62±0.21c |
| | Purple sand pot | 3.06±0.20d | 1.63±0.01b | 17.71±0.22b | 0.54±0.05bc | 24.47±1.08a | 8.86±0.06e | 26.19±0.62b | 9.77±0.21b | 8.55±0.01b | 155.44±2.02d |
| oolong tea | Tin pot | 11.29±0.02c | 7.42±0.17bc | 0.70±0.02c | 0.51±0.01c | 4.93±0.15c | 10.16±0.51b | 0.60±0.00e | 2.16±0.05c | 0.51±0.04a | 41.46±2.14bc |
| | Glass pot | 9.90±0.26d | 7.43±0.06bc | 0.67±0.00c | 0.36±0.00c | 4.63±0.06d | 3.54±0.00d | 0.78±0.08c | 0.49±0.03d | 0.03±0.00c | 37.94±0.08cd |
| | Pottery pot | 9.82±0.25d | 7.84±0.02b | 0.72±0.01d | 0.33±0.00c | 4.27±0.03e | 5.77±0.08c | 0.83±0.01b | 2.57±0.02b | 0.51±0.00a | 33.60±0.11d |
| | Porcelain pot | 17.22±0.37a | 9.89±0.15a | 1.14±0.05a | 0.74±0.01a | 7.13±0.11a | 11.87±0.63a | 0.97±0.05a | 4.08±0.06a | 0.56±0.01a | 54.10±3.81a |
| | Purple sand pot | 13.52±0.53b | 7.01±0.40c | 0.86±0.01b | 0.49±0.05b | 5.95±0.03b | 9.40±0.02b | 0.64±0.00d | 2.11±0.00c | 0.41±0.04b | 45.67±4.50b |
| dark tea | Tin pot | 2.54±0.14b | 4.70±0.04e | 2.17±0.06c | 0.54±0.02d | 0.46±0.01e | 0.45±0.04d | 0.21±0.00e | 0.32± 0.02b | 0.18±0.00c | 0.78±0.01c |
| | Glass pot | 2.34±0.10b | 5.73±0.29d | 1.76±0.02d | 0.79±0.03c | 0.65±0.06d | 0.49±0.01d | 0.26±0.01d | 0.58±0.02a | 0.24±0.01b | 0.53±0.01d |
| | Pottery pot | 3.47±0.17a | 6.32±0.08c | 2.38±0.07c | 1.25±0.12b | 0.86±0.01c | 0.61±0.00c | 0.28±0.01c | 0.57±0.01a | 0.26±0.01a | 1.16±0.01b |
| | Porcelain pot | 3.55±0.08a | 9.70±0.05a | 4.30±0.31a | 1.47±0.01a | 1.11±0.01b | 0.72±0.01b | 0.40±0.00b | 0.62±0.01a | 0.27±0.00a | 1.65±0.01a |
| | Purple sand pot | 3.78±0.17a | 7.22±0.04b | 3.50±0.00b | 1.38±0.00ab | 6.38±0.05a | 0.95±0.01a | 1.29±0.01a | 0.36±0.04b | 0.05±0.00d | 0.29±0.01e |

**Table S3.** Weight of evaluation factors for each tea category (%).

| Tea | Appearance (*a*) | Infusion color (*b*) | Aroma (*c*) | Taste (*d*) | Leaf residue (e) |
|---|---|---|---|---|---|
| Green tea | 25 | 10 | 25 | 30 | 10 |
| Black tea | 25 | 10 | 25 | 30 | 10 |
| Oolong tea | 20 | 5 | 30 | 35 | 10 |
| Dark tea | 20 | 15 | 25 | 30 | 10 |
| White tea | 25 | 10 | 25 | 30 | 10 |
